# The Antecedents and Buffer of Social Media Fatigue: A Moderating Role of Dispositional Mindfulness

**DOI:** 10.1002/pchj.70062

**Published:** 2025-10-30

**Authors:** Xue Yao, Junzhe Zhao, Hang Zhang, Wenfan Chao, Minghui Wang

**Affiliations:** ^1^ School of Psychology, Henan University Kaifeng People's Republic of China

**Keywords:** dispositional mindfulness, fear of missing out, information overload, perceived stress, social media fatigue

## Abstract

Social media fatigue negatively affects users' cognitive, emotional, and behavioral faculties. Therefore, the identification of risk factors associated with this phenomenon is essential for the development of preventative measures against social media fatigue. This study aimed to explore the relationship between fear of missing out and social media fatigue, the mediating role of information overload and perceived stress, and the moderating role of dispositional mindfulness. Adopting a longitudinal cluster sampling design, this study assessed college students using several psychometric instruments: Fear of missing out scale, information overload scale, Chinese perceived stress scale, social media fatigue scale, and mindfulness attention awareness scale. Data from 743 college students, collected and matched across three‐time points, were analyzed to test the mediation and moderation effects. Findings from the study indicated that the independent and chain mediating effects of information overload and perceived stress were significant. Moreover, the negative moderating influences of dispositional mindfulness were also found to be significant. The results suggest that fear of missing out influences social media fatigue through two parallel pathways—information overload and perceived stress—and through a serial pathway involving both variables. Dispositional mindfulness can mitigate the impact of fear of missing out on information overload or perceived stress, as well as alleviate the mediating role of information overload and perceived stress. These findings provide valuable insights into social media fatigue and have significant implications for its prevention and intervention.

## 1 Introduction

### 1.1 Research Background

In recent years, a wide variety of social media platforms (SMPs) have proliferated, establishing themselves as essential conduits for the dissemination and acquisition of information, entertainment, and social interaction. For instance, a study by the Pew Research Center (2025) found that 45% of teenagers think that they spend too much time on social media. A report in 2025 stated that China has nearly 1.123 billion Internet users, with mobile Internet users reaching as high as 1.116 billion. Most Internet users are concentrated among the middle‐aged and young groups (China Internet Network Information Center 2025). As access to multifarious SMPs has become increasingly accessible and the demand for online communication and information acquisition is growing, social media is favored by more individuals, especially the younger generation. The proportion of teenagers using social media every day is as high as 84% (Price et al. 2022). Especially during the COVID‐19 pandemic, the time teenagers spend on social media nearly doubled (Samji et al. 2021), and the consumption of social media news increased by nearly 47% (Anand et al. 2021). The intensity of social media usage can positively predict social media fatigue (SMF) (Malik et al. 2021), which refers to the mental exhaustion that users feel in the process of using social media. It is a negative emotional experience like boredom and tiredness (Dhir et al. 2018). For users, SMF is often accompanied by anxiety and depression (Dhir et al. 2018), and it can also have an impact on academic performance decrement (Malik et al. 2021). For SMPs, SMF often leads to negative usage behavior among users (Ou et al. 2023), accompanied by user churn. In other words, this reduced engagement is accompanied by the emergence of fatigue and anxiety, leading some individuals to disengage from SMPs entirely (Zhang 2020). Therefore, it is of great significance to study the antecedents of SMF.

### 1.2 Literature Review

According to Maslow's Hierarchy of Needs, excessive use of social media may be due to the fact that an individual's need for love and belonging has not been adequately met. Therefore, individuals tend to use social media to compensate for this psychological need. Fear of Missing Out (FoMO) can be understood as a self‐regulation predicament resulting from the long‐term lack of psychological needs (Przybylski et al. 2013). Meanwhile, a large number of related studies have demonstrated that FoMO is a principal motivator behind users' high levels of engagement with SMPs (Rosen et al. 2018; Wolniewicz et al. 2017). Therefore, this study investigates FoMO as a key antecedent of social media fatigue. FoMO is a diffuse anxiety caused by being afraid of leaving out other people's exciting experiences or other positive events (Przybylski et al. 2013). Individuals often harbor a desire to remain informed about the experiences of their peers, which drives them to continuously browse SMPs to learn about the activities of others. In this process, the frequency and duration of social media usage increase, consequently heightening their susceptibility to fatigue. Previous research has consistently shown a positive correlation between the FoMO and SMF (Tandon et al. 2021; Song et al. 2023). While research has established a link between FoMO and SMF, the underlying mechanism of this relationship remains largely unexplored.

Previous study has shown that information overload is a significant mediator between FoMO and SMF among Snapchat users, but not among Instagram users (Hattingh et al. 2022). The mediating role of information overload will be reexamined from a cognitive perspective in this study, thereby contributing to a deeper understanding of its impact. Information overload means that a large amount of information received by an individual from social media exceeds the individual's capacity to process information (Zhang et al. 2016). Multiple studies have shown that information overload is one of the main causes of SMF (Xiao et al. 2019; Yu et al. 2018). Individuals whose FoMO is high stay connected to their social networks through social media to meet their cognitive and emotional needs (Gezgin et al. 2017; Stead and Bibby 2017). This constant connection exposes a large amount of information, while the Limited Capacity Model suggests individuals possess finite psychological resources (Lang 2000). Therefore, it can lead to information overload when information exceeds the individual's processing capacity (Eppler and Mengis 2004). Based on the Limited Capacity Model and previous studies, this study believes that individuals with FoMO will constantly check social media and pay attention to what is happening in the outside world. Once the information received exceeds an individual's cognitive ability to process it, the individual will perceive information overload, which will in turn lead to social media fatigue. Therefore, this study proposes:

*Information overload acts as a mediator between FoMO and SMF*.

In addition to cognitive pathways, FoMO likely impacts social media fatigue through an emotional mechanism. This study posits that FoMO functions as an emotional stressor, elevating users' perceived stress, which in turn contributes to fatigue. Accordingly, this study proposes perceived stress as a second key mediator. Perceived Stress refers to individual's perception and evaluation of stressful events, which determines whether and to what degree those events can cause stress (Ge et al. 2021). When a person leaves the online world, he/she will worry about losing significant messages (Barry and Wong 2020), and the fear of lacking information can cause restlessness, pressure, and dejection (Rozgonjuk et al. 2021). This emotional stress is posited to contribute significantly to fatigue. In addition, according to the Limited Capacity Model, an individual's mental resources are limited (Lang 2000). Therefore, when the perceived stress by an individual exceeds their processing capacity, the user will feel exhausted and powerless. Humans desire beneficial experiences (Vargas et al. 2019) and often feel anxious, depressed, and upset when their friends engage in these experiences while they do not (Akat et al. 2022). The stress induced by this experience is a plausible antecedent to SMF. Therefore, this study proposes:

*Perceived stress mediates the relationship between FoMO and SMF*.

Supporting this link, Misra and Stokols (2012) found that information overload is a significant positive predictor of perceived stress among college students. Users' constant contact with social media due to the FoMO creates a flood of information, leading to overload that is related to stress (Lee, Lee, and Suh 2016). This study suggests that the FoMO may lead to information overload among high‐use social media users (such as college students), whose social connections are bombarded with too much information, which challenges their processing abilities and causes stress. As a result, these social media users may experience adverse outcomes such as fatigue (Tandon et al. 2021; Dhir et al. 2018). Therefore, this study proposes:

*Information overload and perceived stress sequentially mediate the relationship between FoMO and SMF*.

Having established the detrimental pathways from FoMO to social media fatigue, the focus now shifts to identifying protective factors that may buffer this relationship. The Mindfulness Stress Buffering Account (Creswell and Lindsay 2014) provides a compelling theoretical framework for this inquiry. This theory suggests that dispositional mindfulness can mitigate the negative consequences of stressors, thereby alleviating outcomes such as fatigue. Thus, this study posits that dispositional mindfulness acts as a crucial moderator in the FoMO–SMF link. Dispositional Mindfulness is an individual trait referring to one's habitual awareness (Baer et al. 2004). It facilitates individuals to pay attention to their current emotions and awareness, and this cognitive style is conducive to promoting individuals to perceive and accept their bad state (Randal et al. 2015). According to Sun and Liu (2020), undergraduates with higher dispositional mindfulness exhibit superior self‐regulation, as they are better able to alter their thoughts and promptly detect internal state changes. This heightened awareness, in turn, allows them to more readily recognize the negative effects of their behaviors and undertake deliberate actions to avoid adverse consequences. College students with high dispositional mindfulness exhibit greater awareness of their own internal states (Kabat‐Zinn 2003). This self‐awareness enables them, particularly when experiencing tension, to shift their attentional focus from external social information toward their own immediate tasks (Borders et al. 2010). Such attentional regulation serves as a key mechanism for reducing information overload. Furthermore, separate research has directly linked higher dispositional mindfulness with lower levels of perceived stress (Brown and Ryan 2003). Therefore, this study proposes:

*Dispositional mindfulness negatively moderates the role of FoMO on perception of information overload (a) and perceived stress (b)*.

The Mindfulness Stress Buffering Account (Creswell and Lindsay 2014) suggests that mindfulness can mitigate the negative consequences of stressors, promoting overall well‐being. Applying this framework, this study infers that mindfulness may also buffer the adverse effects of FoMO. Specifically, individuals with high dispositional mindfulness tend to focus more on the present moment rather than being excessively preoccupied with the activities of others. Moreover, recent research indicates they possess enhanced information processing capabilities (Zhang et al. 2023). Consequently, even when exposed to substantial information, they are less susceptible to cognitive overload and the resulting fatigue. Furthermore, dispositional mindfulness is associated with more adaptive emotion regulation skills (Dundas et al. 2013). Individuals with high levels of mindfulness are more likely to accept their emotions non‐judgmentally (Bai and Yue 2013; Ulmer et al. 2010), rather than being driven by them into maladaptive behaviors such as social media overuse. This emotional regulation, in turn, can reduce the excessive intake of information and the perception of pressure, thereby alleviating SMF. Supporting this, studies have shown that highly mindful individuals experience fewer negative emotions in response to perceived stress (Xu et al. 2017, 2018), further highlighting mindfulness's role as a buffer. Therefore, this study proposes:

*Dispositional mindfulness negatively moderates the mediating role of information overload (a) and perceived stress (b) between FoMO and SMF*.

Focusing on college students—a primary demographic of social media users—this study investigates the internal mechanisms and boundary conditions of FoMO's influence on SMF, aiming to generate insights that can inform strategies for enhancing mental health and promoting healthy social media use.

### 1.3 Research Model Establishment

According to the Limited Capacity Model (Lang 2000), people have only limited mental resources to process information. The FoMO internally and externally motivates users to engage in compulsive social media monitoring. The cognitive effort required to constantly follow social media drains energy and leads to SMF—the subjective and negative feeling of fatigue caused by social media use (Ravindran et al. 2014). The Limited Capacity Model helps explain how FoMO leads to SMF through two distinct pathways. On the one hand, a cognitive pathway is initiated. FoMO compels individuals to engage in constant social media monitoring, leading to a massive influx of information. According to the Limited Capacity Model, which posits that individuals have finite cognitive resources, this state of information overload depletes their processing capacity, resulting directly in the mental exhaustion characteristic of SMF. On the other hand, a parallel emotional pathway exists. FoMO itself can be appraised as a psychological stressor, triggering a stress response. This emotional strain, much like cognitive overload, consumes the same limited pool of psychological resources, thereby contributing to fatigue.

The Mindfulness Stress Buffering Account (Creswell and Lindsay 2014) provides the theoretical basis for the proposed moderating effect of dispositional mindfulness. According to the Mindfulness Stress Buffering Account, mindfulness can reduce individuals' destructive evaluation of stressful events, reduce stress response, and further promote physical and mental health. Given that dispositional mindfulness is known to alleviate negative emotions arising from general life stress (Xu et al. 2018), it is plausible that this buffering effect extends to specific digital stressors like FoMO. As a stressor, FoMO can contribute to SMF through two primary pathways: by fostering a state of information overload and by heightening perceived pressure. However, individuals high in dispositional mindfulness are characterized by present‐moment awareness and a non‐judgmental stance. When encountering cues that trigger FoMO, they are less likely to appraise these cues as highly threatening to their social standing or well‐being. This altered primary appraisal reduces the initial stress reaction. Consequently, they are less compelled to engage in compensatory behaviors like compulsive browsing, which conserves cognitive resources and prevents the chain reaction that leads to information overload and perceived stress. Thus, mindfulness is theorized to weaken the positive relationships between FoMO and its mediators, ultimately mitigating SMF.

By integrating the Limited Capacity Model and the Mindfulness Stress Buffering Account, this study establishes a comprehensive theoretical framework to address a limitation in existing literature. While prior research has seldom examined the underlying mechanisms and boundary conditions between FoMO and SMF, this investigation advances the field by elucidating the cognitive pathway through which FoMO depletes mental resources and leads to fatigue, as explained by the Limited Capacity Model. Furthermore, it introduces dispositional mindfulness as a critical regulatory factor, grounded in the Mindfulness Stress Buffering Account, which buffers this detrimental pathway by altering cognitive appraisal of FoMO triggers, thereby conserving resources and mitigating negative outcomes. This theoretical integration not only helps clarify the influencing mechanisms between FoMO and SMF from a cognitive perspective but also deepens the discourse on boundary conditions, offering valuable insights for developing interventions aimed at alleviating SMF. Thus, the study provides a nuanced understanding of the interplay between a risk factor and a protective resource, contributing meaningfully to the evolving research on SMF.

## 2 Method

### 2.1 Participants and Procedure

In this study, a cluster sampling method was used to collect data. Participants were recruited from four universities in a central province of China. A multi‐time survey was used to collect data. Questionnaires were distributed offline to the subjects at three time points, with a one‐month interval between the time points. At the initial time point (T1), participants provided demographic information and completed the FoMO scale and the mindfulness attention awareness scale. At the second time point (T2), they were required to fill in identifiers (student number and name) along with the information overload scale and Chinese perceived stress scale. Finally, at time point 3 (T3), participants again entered their identifiers and completed the SMF scale. This study has obtained the consent and cooperation of counselors or teachers in advance, and the experimenter learned about the students from the teachers. Prior to administering the questionnaire, researchers informed the participants about the study's purpose, procedures, and the voluntary nature of their involvement. Participants were assured that their responses would be kept confidential, used solely for academic purposes, and would not affect their academic standing. Participants were informed that they could withdraw at any time without penalty.

Data were collected at three time points, demonstrating high retention rates throughout the study. Initially, 1169 questionnaires were distributed at Time 1, yielding 1097 valid responses (93.84% effective rate). At Time 2, 995 valid responses were collected and successfully matched with 902 participants from Time 1. Finally, the Time 3 data collection yielded 970 valid responses, resulting in a final matched sample of 743 participants for the longitudinal analysis. All participants met the study's inclusion criteria; therefore, no one was excluded. The baseline variables of the matched samples and the lost samples were compared, and no significant difference was found (*p* = 0.244), supporting the hypothesis that the data were MCAR. Among these 743 matched data, the minimum age is 16 and the maximum age is 24 (M = 19.764, SD = 1.168); 332 were males (44.68%) and 411 were females (55.32%); 135 were only‐children (18.17%) and 608 were not (81.83%). Regarding the places of birth, 251 participants were from urban areas (33.78%) and 492 from rural areas (66.22%). By academic year, 465 were sophomores (62.58%), 168 juniors (22.61%), and 110 seniors (14.81%).

### 2.2 Measures

#### 2.2.1 Fear of Missing out Scale

This scale was originally compiled by Przybylski et al. (2013) and subsequently adapted by Li et al. (2019) to suit the Chinese context. It comprises eight items, with two dimensions (fear of missing information and fear of missing situations). The scale uses a 5‐point Likert scale, ranging from 1 to 5. All items are scored in a positive way, with an overall score ranging from 8 to 40 points. The higher the total score, the higher the level of the fear of missing out. In this study, the Cronbach's *α* of this scale was 0.789.

#### 2.2.2 Information Overload Scale

This scale was adapted by Huang (2020) from the original scale compiled by Zhang et al. (2016). The scale has four items in one dimension. The scale uses a 5‐point Likert scale, ranging from 1 to 5, all of which are positive scores, with an overall ranging score from 4 to 20 points. The higher the total score, the higher the level of information overload. In this study, the Cronbach's *α* of this scale was 0.815.

#### 2.2.3 Chinese Perceived Stress Scale

The scale was originally compiled by Cohen et al. (1983) and later revised by Yang and Huang (2003). It has 14 items, including two dimensions of Tension and Out of Control. The scale uses a 5‐point Likert scale, ranging from 0 to 4, and includes items scored in reverse (the dimension of out of control), with an overall score ranging from 0 to 56. A higher total score indicates a higher level of perceived stress. In this study, the Cronbach's α of this scale was 0.683.

#### 2.2.4 Social Media Fatigue Scale

This scale was translated and revised by Li et al. (2022) from the original scale developed by Lee, Son, and Kim (2016). The scale is a single dimension with 5 items. The scale uses a 5‐point Likert scale ranging from 1 to 5, with an overall score ranging from 5 to 25 points. A higher overall score indicates greater social media fatigue. In this study, the Cronbach's *α* of this scale was 0.873.

#### 2.2.5 Mindfulness Attention Awareness Scale

The scale was revised by Chen et al. (2012). The scale has a single dimension and a total of 15 items. The scale uses a six‐point Likert scale ranging from 1 to 6, with an overall score ranging from 15 to 90. A higher score indicates a higher level of dispositional mindfulness. In this study, the Cronbach's *α* of this scale was 0.876.

#### 2.2.6 Control Variables

Several demographic and socio‐economic factors were controlled for, including school, place of origin, gender, only‐child status, and academic grade.

### 2.3 Statistical Analysis

Prior to the main statistical analysis, the data was carefully screened and cleaned. First, missing demographic information was verified and, where possible, completed by consulting supervising teachers. Following this, cases with substantial missing data or extreme outliers were removed. For the remaining data, occasional missing values were handled using the outliers were minorized to the 99th percentile to reduce their influence.

All statistical analyses were conducted using SPSS version 26.0. The analysis proceeded in two stages. First, this study calculated descriptive statistics and Pearson correlation coefficients for all study variables. Second, with FoMO as the predictor and SMF as the outcome variable, the mediating role of information overload and perceived stress and the moderating role of trait mindfulness were examined.

### 2.4 Test of Common Method Biases

The result of Harman's one‐factor test showed the variance explained by the first factor was 17.68%, which is below 40% (Zhou and Long 2004). Thus, the results suggested that common method bias does not pose a substantial concern in this study.

## 3 Results

### 3.1 Descriptive Statistics and Correlation Analysis

As shown in Table 1, all study variables were significantly intercorrelated in the expected directions. Specifically, FoMO, information overload, and perceived stress were all positively associated with each other and with SMF. In contrast, dispositional mindfulness exhibited a significant negative relationship with all other variables.

**TABLE 1:** Descriptive statistics and correlation analysis.

	M	SD	1	2	3	4
1. Fear of missing out T1	20.498	5.811	1.000			
2. Information overload T2	10.834	3.335	0.197***	1.000		
3. Perceived stress T2	25.626	5.202	0.215***	0.296***	1.000	
4. Social media fatigue T3	14.062	4.159	0.197***	0.459***	0.257***	1.000
5. Dispositional mindfulness T1	63.536	10.934	−0.164***	−0.236***	−0.349***	−0.212***

*** *p* < 0.001.

### 3.2 Tests of the Mediating Effect

Hierarchical regression analysis was employed to test the mediating effects, with results detailed as follows. FoMO was found to significantly predict SMF (*b* = 0.056, SE = 0.024, *p* = 0.021), information overload (*b* = 0.086, SE = 0.021, *p* < 0.001), and perceived stress (*b* = 0.151, SE = 0.032, *p* < 0.001), all positively. Additionally, information overload significantly and positively predicted perceived stress (*b* = 0.407, SE = 0.056, *p* < 0.001) and SMF (*b* = 0.488, SE = 0.043, *p* < 0.001). Perceived stress also significantly positively predicted SMF (*b* = 0.084, SE = 0.027, *p* = 0.002).

In this study, the mediating effects were examined by the bootstrap method, with results presented in Table 2. It was found that FoMO influenced SMF through information overload (95% CI = [0.019, 0.068]). Additionally, FoMO influenced SMF through perceived stress (95% CI = [0.003, 0.025]). Moreover, information overload and perceived stress played a chain mediating role between FoMO and SMF (95% CI = [0.001, 0.006]). As none of these confidence intervals included 0, H1, H2, and H3 were supported.

**TABLE 2:** Test of mediated effects.

Model pathways	Effect size	LLCI	ULCI
Total effect	0.058	0.031	0.086
Indirect effect 1: Fear of missing out T1 → Information overload T2 → Social media fatigue T3	0.042	0.019	0.068
Indirect effect 2: Fear of missing out T1 → Perceived stress T2 → Social media fatigue T3	0.013	0.003	0.025
Indirect effect 3: Fear of missing out T1 → Information overload T2 → Perceived stress T2 → Social media fatigue T3	0.003	0.001	0.006

*Note:* Bootstrapping sample = 5000.

### 3.3 Test of the Moderating Effect

**FIGURE 1:**
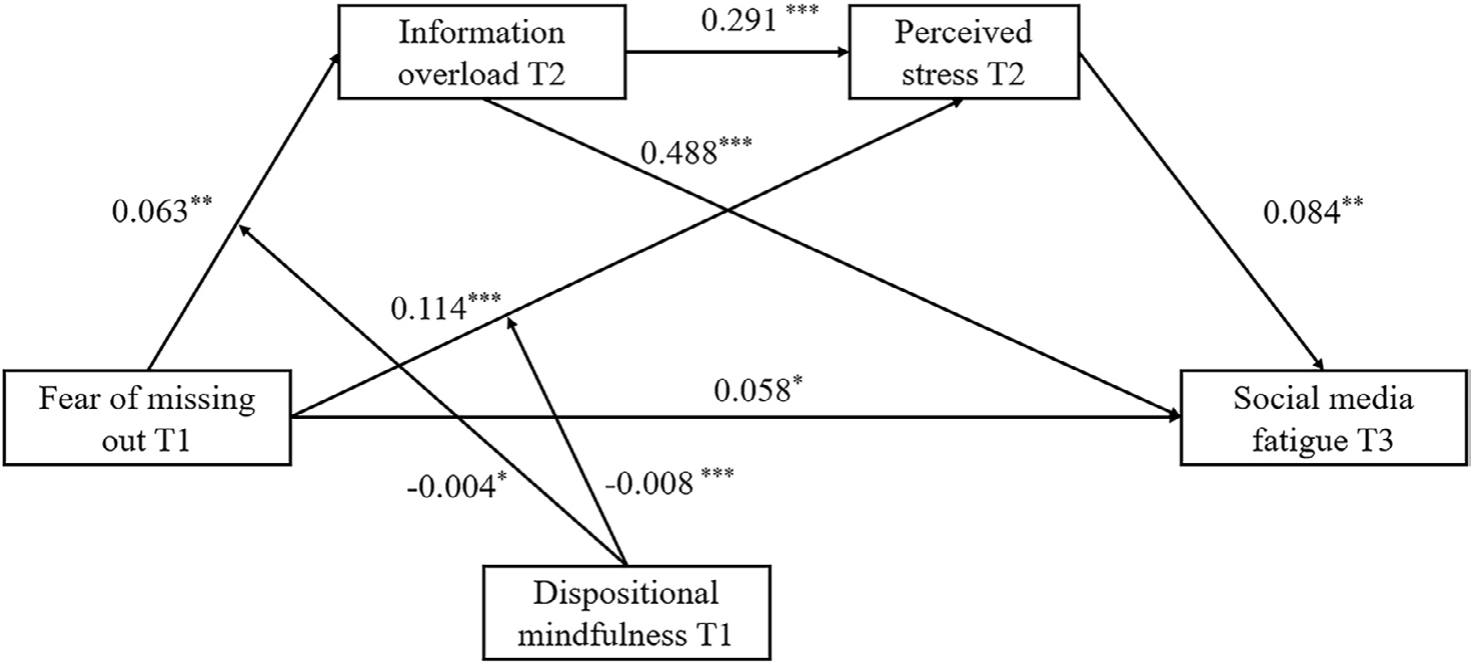
Results of the study model.

Results from the moderation analysis indicated significant negative effects of the interaction terms between FoMO and dispositional mindfulness on both information overload (*b* = −0.004, SE = 0.002, *p* = 0.013) and perceived stress (*b* = −0.008, SE = 0.002, *p* < 0.001). Consequently, hypothesis 4a and hypothesis 4b were verified. The path coefficient for each interaction was depicted in Figure 1.

**FIGURE 2:**
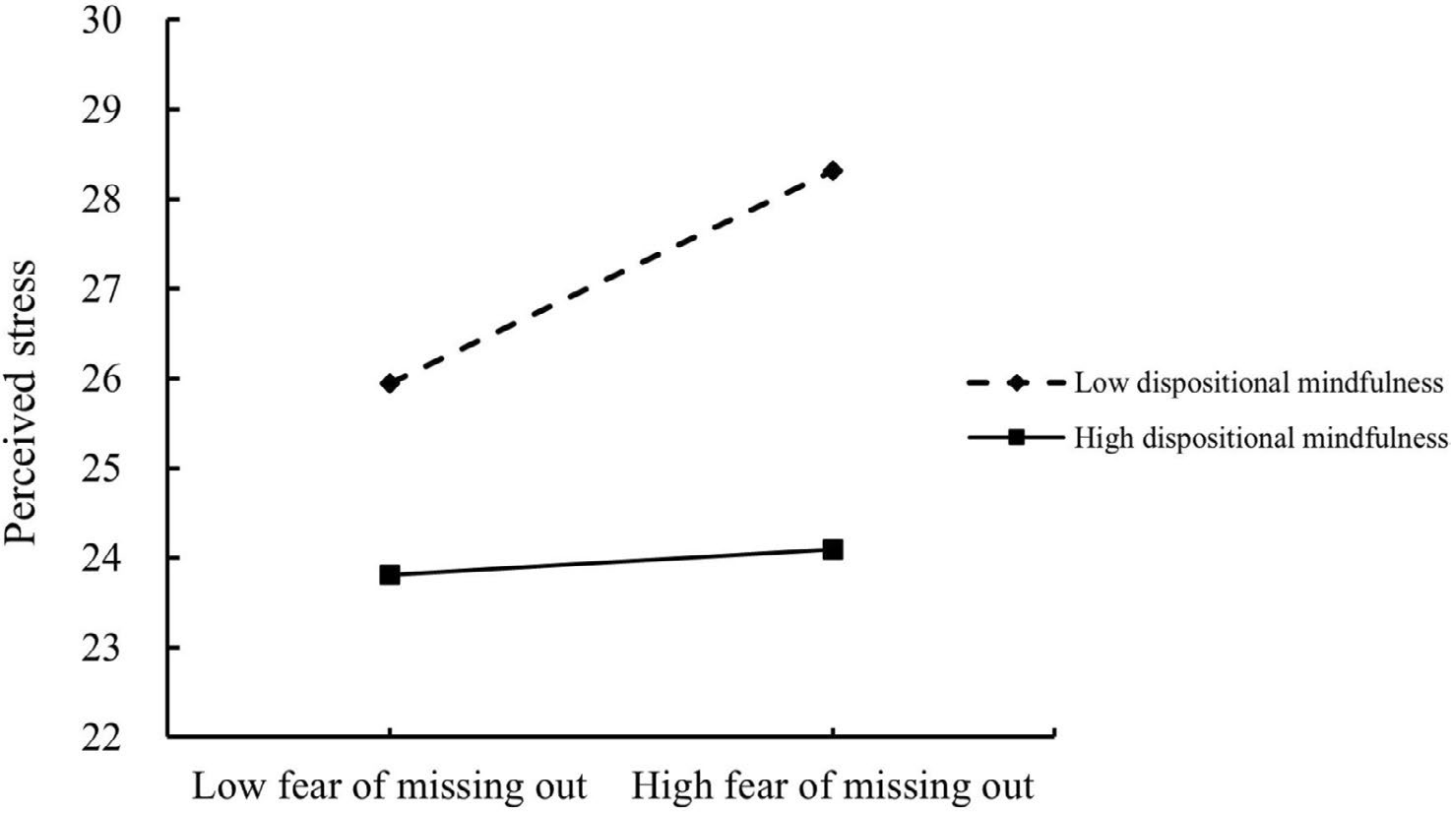
Moderating effect on information overload. Moderating effect plot reflects the moderating effect of dispositional mindfulness on the relationship between fear of missing out and information overload.

**FIGURE 3:**
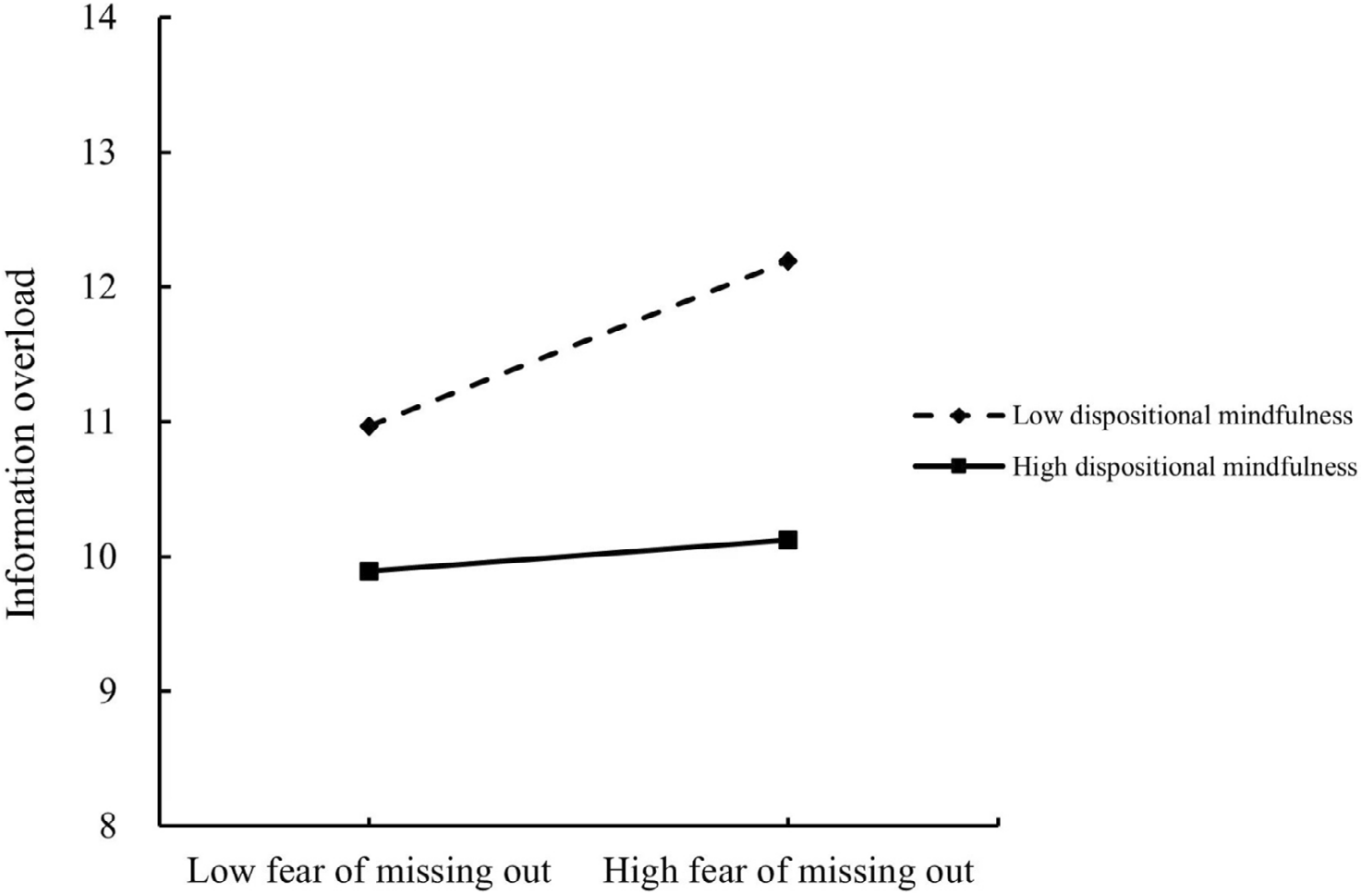
Moderating effect on perceived stress. Moderating effect plot reflects the moderating effect of dispositional mindfulness on the relationship between fear of missing out and perceived stress.

In this study, a simple slope analysis was used to further analyze the moderating effects of dispositional mindfulness on the relationships between FoMO and both information overload and perceived stress. The results, depicted in Figure 2, demonstrated that at a low level of dispositional mindfulness, FoMO significantly impacted information overload (simple slope = 0.106, *t* = 3.909, *p* < 0.001, 95% CI = [0.053, 0.159]). Conversely, at a high level of dispositional mindfulness, this effect was not significant (simple slope = 0.020, *t* = 0.728, *p* = 0.467, 95% CI = [−0.033, 0.073]), thus reconfirming hypothesis 4a. Similarly, as shown in Figure 3, at a low level of dispositional mindfulness, FoMO significantly influenced perceived stress (simple slope = 0.204, *t* = 4.987, *p* < 0.001, 95% CI = [0.123, 0.284]). However, at a high level of dispositional mindfulness, the influence was not significant (simple slope = 0.024, *t* = 0.604, *p* = 0.546, 95% CI = [−0.055, 0.104]), thereby reconfirming hypothesis 4b.

In this study, Process Model 84 was employed to test the moderated mediation effects. When information overload served as the mediating variable, the moderated mediating effect was −0.002, with a 95% CI that did not contain 0, thereby confirming H5a. Similarly, when perceived stress was the mediating variable, the moderated mediating effect was −0.001, with a 95% CI that also did not contain 0, confirming H5b.

Detailed findings presented in Table 3 revealed that the mediation effect of information overload was significant at a low level of dispositional mindfulness (effect size = 0.052, SE = 0.014, 95% CI = [0.024, 0.081]), but not at a high level (effect size = 0.010, SE = 0.015, 95% CI = [−0.019, 0.039]). For the mediating variable of perceived stress, the mediation effect was significant when the level of dispositional mindfulness was low (effect size = 0.017, SE = 0.007, 95% CI = [0.005, 0.031]), and not significant when the level of dispositional mindfulness was high (effect size = 0.002, SE = 0.004, 95% CI = [−0.006, 0.012]). These results collectively confirmed hypotheses 5a and 5b.

**TABLE 3:** Test of moderated mediation model.

Mediator	Moderator	Effect size	SE	LLCI	ULCI
Information overload T2	Low dispositional mindfulness	0.052	0.014	0.024	0.081
	High dispositional mindfulness	0.010	0.015	−0.019	0.039
	Difference between groups	−0.042	0.019	−0.080	−0.006
Perceived stress T2	Low dispositional mindfulness	0.017	0.007	0.005	0.031
	High dispositional mindfulness	0.002	0.004	−0.006	0.012
	Difference between groups	−0.015	0.007	−0.030	−0.002

*Note:* Bootstrapping sample = 5000.

## 4 Discussion

The demands of academic and social life mean that college students often exhibit a heightened need for social interaction, and web‐based social media is an important part of their social life (Ping et al. 2012). Meanwhile, college students are at an age of curiosity and willingness to explore, eager to know the wonderful experiences of others and establish connections with them; that is, they are more prone to FoMO (Chai et al. 2018; Przybylski et al. 2013). Therefore, the results of this study will be interpreted in the unique development and environmental context of college students.

### 4.1 The Mediating Role of Information Overload and Perceived Stress

Initially, the results demonstrated that information overload mediated the relationship between FoMO and SMF, validating H1. This result was consistent with prior research, which has also indicated that information overload positively influences SMF (Xiao et al. 2019; Yu et al. 2018). The FoMO can be attributed to deficiencies in an individual's need for love and belonging. Individuals experiencing high levels of FoMO may engage with social media to compensate for these unmet psychological demands. Consequently, these individuals are often driven to continuously gather and process a wide array of information on SMPs (Song et al. 2023). Information overload occurs when the amount of information an individual receives exceeds their processing capacity (Eppler and Mengis 2004). This overwhelming influx of information can lead to cognitive overload, ultimately resulting in fatigue. The individual's own resources are limited, and excessive information will consume the individual's cognitive and time resources, making them too tired to cope effectively, thus causing SMF.

In addition, our findings further suggested perceived stress mediated the relationship between FoMO and SMF, thereby confirming H2. Cohen et al. (1983) argued that perceived stress occurred when people perceived a situation to be threatening or otherwise demanding and lacked sufficient resources to deal with the situation. Consequently, when individuals with high levels of FoMO are unable to satisfy their social and emotional needs through social media, they are likely to experience elevated perceived stress. This heightened stress may, in turn, trigger a maladaptive coping mechanism, such as compensatory social media overuse (Jiang et al. 2017). Such prolonged engagement can impair individuals' ability to manage their time effectively, ultimately depleting their cognitive resources and contributing to social media fatigue.

Furthermore, the results supported a chain mediation model (H3), indicating that FoMO influences SMF sequentially through information overload and then perceived stress. This finding complements the parallel mediation results, suggesting these variables act both individually and serially to explain the overall effect.

In conclusion, this study conducted the survey using relevant scales that fit the Chinese context, ensuring the accuracy of the language and the relevance of the culture, enhancing the validity of the research results in the Chinese population, and providing a relatively more accurate model. While the identified mediating pathways of information overload and perceived stress are consistent with findings from Western studies, their salience and intensity may be amplified within the Chinese cultural context. The collectivist nature of Chinese society, which emphasizes group belonging and social comparison, can heighten both fear of social exclusion and the pressure to remain constantly informed, thus potentially strengthening these mediation effects. This cultural amplifier implies that the compulsive monitoring behavior driven by FoMO may have more obvious information overload and subsequent fatigue experience compared with an individualistic society. Furthermore, when individuals feel that they have failed to meet these social expectations through social media, it may increase their perceived stress. Therefore, the research findings not only confirmed the universal cognitive mechanism of the Limited Capacity Model hypothesis but also emphasize the possible role of cultural background in potentially reinforcing these pathways. While the mediating pathways of information overload and perceived stress are likely applicable to other populations, their intensity and manifestation are arguably heightened in a university setting. The necessity to constantly monitor diverse platforms for both academic and social survival creates a perfect storm for information overload. Similarly, the perceived stress arising from FoMO is compounded by pre‐existing academic pressures, making students particularly susceptible to its negative effects.

### 4.2 The Moderating Role of Dispositional Mindfulness

The results indicated that dispositional mindfulness significantly moderated the predictive effect of FoMO on both information overload and perceived stress. This negative moderation confirmed hypotheses 4a and 4b of the study. In other words, dispositional mindfulness buffered the positive relationship between FoMO and its outcomes. Specifically, the effects of FoMO on both information overload and perceived stress were significant only at low levels of dispositional mindfulness but became non‐significant at high levels. Higher dispositional mindfulness means individuals have a clearer understanding of their own experiences (including physical sensations, thoughts, emotions, etc.) (Kabat‐Zinn 2003), which makes them focus on the current task in the presence of external distractions (Borders et al. 2010) and thus avoid exposure to a large amount of information, reducing information overload. Moreover, individuals with high levels of dispositional mindfulness exhibit enhanced attentional stability, enabling them to absorb and process a broader range of information more effectively (Zhang et al. 2023). Therefore, even when exposed to large volumes of information, they are less likely to experience information overload. Meanwhile, higher dispositional mindfulness indicates more acceptance of emotions (Ulmer et al. 2010), which contributes to individuals' ability to mitigate perceived stress.

Finally, the study revealed that the moderator of dispositional mindfulness on above indirect effects, which included indirect effects of FoMO on SMF via information overload and perceived stress. This finding suggested that dispositional mindfulness significantly and negatively moderates the mediating roles of both information overload and perceived stress. Specifically, at low levels of dispositional mindfulness, information overload and perceived stress more strongly mediate the relationship between FoMO and SMF. These results validate hypotheses 5a and 5b. According to the re‐perception model of mindfulness, individuals who attentively and nonjudgmentally process their internal and external experiences can induce a transformative shift in their cognitive style. This shift enables them to recognize the transience of negative emotions, reducing their habitual responses to immediate emotional stimuli. Consequently, this awareness fosters a higher tolerance for experiencing unpleasant internal emotions (Bai and Yue 2013), facilitating greater emotional resilience and stability. Therefore, among college students with high levels of dispositional mindfulness, there is an enhanced capacity to tolerate feelings of nervousness and uneasiness, which enables them to avoid exposure to excessive information. Consequently, the mediating effect of information overload becomes less significant. The model also suggests that dispositional mindfulness can avoid individuals' over‐involvement in negative emotions and thus reduce stress (Chesin et al. 2016), and individuals with higher dispositional mindfulness experience less stress. This provides a theoretical basis for the moderating effect of perceived stress at high dispositional mindfulness levels.

Previous studies mainly focused on identifying risk factors (e.g., FoMO), but the findings of this study have shifted the focus to a protective personal trait. The results showed that for individuals with a high level of mindfulness, the positive path from FoMO to information overload and perceived stress is significantly weakened. This provides empirical support for the Mindfulness Stress Buffering Theory and indicates that mindfulness training can be an effective intervention measure to mitigate the negative psychological impact of social media use, which shifts the narrative from simple problem identification to potential solutions.

### 4.3 Theoretical and Practical Implications

Using an undergraduate sample, this study elucidates the mechanisms and boundary conditions of FoMO's influence on SMF, thereby making a significant contribution to the social media fatigue literature. SMF is a key indicator of the mental health of contemporary college students. Therefore, investigating its antecedents is crucial for developing interventions to promote student well‐being. This study examined the mediating role of information overload and perceived stress from a cognitive perspective, broadening the research perspective and providing theoretical reference for subsequent research in related fields. Another key theoretical contribution of this study is the determination that dispositional mindfulness is a crucial buffer against the development of SMF. In summary, the integration of the Limited Capacity Model and the Mindfulness Stress Buffering Account provides a powerful, dual‐process framework for understanding these results. The Limited Capacity Model successfully explains the maladaptive cognitive pathway: FoMO depletes finite attentional resources, leading to overload, stress, and fatigue. The Mindfulness Stress Buffering Account, conversely, illuminates the regulatory process: dispositional mindfulness acts as a protective resource, buffering this pathway at the initial appraisal stage by reducing the threat value of FoMO triggers and conserving cognitive capacity. This integrated model offers a more comprehensive theoretical explanation than either theory alone, moving from descriptive correlation toward a mechanistic understanding of the interplay between risk and protective factors in SMF. These insights not only advance the theoretical but also provide a clear foundation for practical interventions aimed at mitigating these negative effects through mindfulness‐based training.

The findings of this study offer important implications for educational institutions and relevant stakeholders to develop targeted interventions. To effectively address SMF among university students, this study proposes a multi‐level, systematic framework for practice. At the university level, administrators should incorporate digital literacy education and mindfulness training into institutional programs such as freshman orientation and well‐being curricula to help students develop healthy social media habits from the very beginning. Simultaneously, mental health centers should recognize FoMO as a significant psychosocial stressor, incorporating social media usage screening into routine assessments and offering targeted group counseling that enhances students' self‐worth and real‐life social skills to address the root causes of FoMO. At the individual level, students should be encouraged to develop self‐regulatory skills regarding technology use. Participating in campus mindfulness clubs or using focus training tools can effectively strengthen awareness and control over social media impulses. Students are also advised to establish personalized digital hygiene habits, such as regularly curating their follow lists and setting Do‐Not‐Disturb periods. Finally, SMPs also need to take responsibility for promoting user well‐being by improving recommendation algorithms to reduce exposure to repetitive content and providing more transparent information management tools to collectively foster a healthier digital environment. This collaborative and multi‐faceted intervention model not only helps alleviate current issues related to SMF but also offers a practical pathway for building a healthier digital ecosystem on campus.

## 5 Limitations and Prospects

This study contributes to the literature by elucidating the internal mechanisms and identifying a key boundary condition of the relationship between FoMO and SMF. In doing so, it both expands the current understanding of this phenomenon and provides a foundation for future research. However, this study has several limitations that warrant consideration. Firstly, this study's reliance on a college student sample, while justified by their high social media usage, limits the generalizability of the findings. Future research should therefore replicate these findings in more diverse populations, such as different occupational groups, to enhance external validity. Furthermore, the sample was drawn from a single province in China, which may limit the cultural and regional generalizability of the findings. While this approach enhances sample homogeneity by controlling for regional variables, it also restricts the applicability of the results to a broader population. Future research should therefore aim to recruit a more geographically and culturally diverse sample, ideally using probability sampling methods, to establish the robustness of the current model across different contexts.

Secondly, this study's reliance on self‐report measures raises concerns about potential common method bias. This bias may artificially inflate the observed relationships between variables, thereby threatening the internal validity of our findings. Future research could overcome these limitations by employing experimental or longitudinal designs. Such approaches would allow for a more rigorous examination of the causal relationships between variables, thereby strengthening internal validity. Additionally, incorporating multi‐informant data, such as peer or parent reports, would help to corroborate the self‐reported findings and provide a more comprehensive understanding of the phenomena. In addition, data were collected using multi‐wa sampling without controlling baseline levels of mediating and outcome variables. Future studies may consider long‐term and multi‐frequency follow‐up studies to further explore the changing characteristics of the relationship between different variables.

Finally, the theoretical model of this study has been supported, but there is still much room for exploration regarding the relationships among the variables. While the research model posits a linear relationship between FoMO and information overload, it is theoretically plausible that this relationship is more complex. A certain level of information monitoring driven by FoMO might be adaptive to avoid real‐world losses (e.g., missing important academic updates). It is possible that an inflection point exists, beyond which compulsive monitoring becomes maladaptive and leads to overload. The linear approach provides a valid and parsimonious representation of the relationship within the range of FoMO levels prevalent in the research sample of college students' social media users. However, future research could employ nonlinear models to precisely identify this threshold, which would have significant implications for developing targeted interventions. Despite strong theoretical justification, the cross‐sectional nature of the research data limits definitive causal inferences. Although the conceptual framework in this study positions FoMO as an antecedent to perceived stress, the nature of cross‐sectional data prevents the drawing of definitive causal conclusions. Consequently, the possibility of reverse causality (i.e., stress exacerbating FoMO) or a bidirectional relationship between these two constructions cannot be ruled out. Future longitudinal studies that measure these variables at multiple time points are needed to untangle the complex temporal dynamics between these constructs. It is important to note that while this research positioned dispositional mindfulness as a moderator based on our theoretical focus on buffering mechanisms, it may also be modeled as an antecedent that reduces the initial development of FoMO. These roles are not mutually exclusive. Future research could construct a more comprehensive model where mindfulness serves both as a distal predictor that lowers baseline FoMO levels and a proximal moderator that mitigates the negative consequences of FoMO once it occurs. Testing such a model would require longitudinal data to disentangle these temporal effects.

## Ethics Statement

The questionnaire and methodology for this study were approved by the Ethics Committee of Henan Province Key Laboratory of Psychology and Behavior (Ethics approval number: 20230826001).

## Conflicts of Interest

The authors declare no conflicts of interest.

## Data Availability

The data that support the findings of this study are available on request from the corresponding author. The data are not publicly available due to privacy or ethical restrictions.
